# New Approach to Synthesizing Cathode PtCo/C Catalysts for Low-Temperature Fuel Cells

**DOI:** 10.3390/nano14100856

**Published:** 2024-05-14

**Authors:** Sergey Belenov, Dmitriy Mauer, Elizabeth Moguchikh, Anna Gavrilova, Alina Nevelskaya, Egor Beskopylny, Ilya Pankov, Aleksey Nikulin, Anastasia Alekseenko

**Affiliations:** 1 Faculty of Chemistry, Southern Federal University, 7 Zorge St., Rostov-on-Don 344090, Russia; dima333000@yandex.ru (D.M.); liza.moguchix@mail.ru (E.M.); agavrilo@sfedu.ru (A.G.); alya.nevelskaya@mail.ru (A.N.); gosha200225@yandex.ru (E.B.); an-an-alekseenko@yandex.ru (A.A.); 2 Prometheus R&D LLC, 4G/36 Zhmaylova St., Rostov-on-Don 344091, Russia; 3 Federal Research Center “The Southern Scientific Center of the Russian Academy of Sciences” (SSC RAS), Federal State Budgetary Institution of Science, 41 Chekhova St., Rostov-on-Don 344006, Russia; aynikulin@sfedu.ru; 4 Research Institute of Physical Organic Chemistry, Southern Federal University, 194/2 Stachki St., Rostov-on-Don 344090, Russia; ipankov@sfedu.ru

**Keywords:** catalyst synthesis, PEMFC, electrocatalyst activity, PtCo/C, composite support, oxygen electroreduction, ESA, MEA

## Abstract

The presented study is concerned with a new multi-step method to synthesize PtCo/C materials based on composite Co_x_O_y_/C that combines the advantages of different liquid-phase synthesis methods. Based on the results of studying the materials at each stage of synthesis with the TG, XRD, TEM, SEI, TXRF, CV and LSV methods, a detailed overview of the sequential changes in catalyst composition and structure at each stage of the synthesis is presented. The PtCo/C catalyst synthesized with the multi-step method is characterized by a uniform distribution of bimetallic nanoparticles of about 3 nm in size over the surface of the support, which result in its high ESA and ORR activity. The activity study for the synthesized PtCo/C catalyst in an MEA showed better current–voltage characteristics and a higher maximum specific power compared with an MEA based on a commercial Pt/C catalyst. Therefore, the results of the presented study demonstrate high prospects for the developed approach to the multi-step synthesis of PtM/C catalysts, which may enhance the characteristics of proton-exchange membrane fuel cells (PEMFCs).

## 1. Introduction

A low-temperature fuel cell is an essential component of hydrogen energy, the most important part of which is a catalyst that accelerates reactions at the anodes and cathodes of fuel cells [1]. Among other metals, platinum is known to exhibit the best catalytic activity in the oxygen reduction reaction (ORR) at the cathode of fuel cells [2]. It is noteworthy that platinum is currently used in the form of nanoparticles (NPs) deposited on a highly dispersed carbon support to increase the electrochemically active surface area (ESA) and enhance material stability. To improve a catalyst’s functional characteristics, the alloying of platinum with transition d-metals, such as Co [3,4,5], Cu [6,7,8,9] and Ni [10,11,12,13], is widespread. An increase in the activity of platinum-based electrocatalysts due to doping with d-metals may be associated with the following general factors: a distance decrease in the Pt–Pt bond [14], a change in the energy of platinum d-orbitals [15], or a change in the surface structure [16]. PtCo catalysts are known to be applied by Toyota in the production of fuel cells for the Toyota Mirai [17]. This indirectly indicates the higher commercial prospects of those systems compared with PtCu and PtNi. It should also be pointed out that according to a series of works, PtCo/C catalysts demonstrate high performance when tested in the membrane electrode assemblies (MEAs) of fuel cells [18,19,20]. In [20], based on the results of comparing synthesized PtCo/C catalysts with a commercial Pt/C analog when tested in MEAs, the bimetallic catalysts were shown to exhibit activity values 1.1–1.7 times higher than those of Pt/C catalysts. In [18], platinum–cobalt catalysts reached the target stability characteristics provided for in the US Department of Energy (DOE) recommendations for MEA study.

According to the literature data, the platinum–cobalt systems of a Pt_3_Co composition [21,22,23,24] are commonly considered as being among the most active ones. Nevertheless, a series of articles [15,25] note that the 1:1 and 1:7 Pt:Co ratios may also exhibit high performance. Thus, in [15], the maximum catalytic ORR activity was shown to be a characteristic of a catalyst with an atomic Pt:Co ratio of about 1:7, while in [25], the material with a 1:1 Pt:Co ratio demonstrated the greatest activity in the ORR. A comparison of Pt_3_Co and PtCo bimetallic materials [26] showed that PtCo materials are characterized by a more uniform particle size distribution and, thus, higher activity in the ORR. Therefore, the choice of an optimal PtCo/C catalyst composition is still a question to be addressed, which may be due to the dependence of a catalyst’s functional characteristics on the average size and size distribution, shape and fine structure of bimetallic NPs, as well as the type support used [27,28].

To date, liquid-phase synthesis methods [29,30,31,32,33,34,35,36,37,38,39], including the borohydride [25,33,34], polyol [35,36] solvothermal [37,38] and microwave [39] ones, are among the most commonly used methods to synthesize bimetallic catalysts. In [25], a comparison was made between the polyol synthesis method and synthesis methods where hydrazine and NaBH4 are used as reducing agents to synthesize Pt/C and PtCo/C catalysts. The catalysts synthesized with the borohydride and polyol methods were reported to exhibit the lower ESA values of about 30 m^2^/g and an average crystallite size of 3 nm, whereas for the method using hydrazine, a significant increase in the crystallite size up to 9 nm and a decrease in the ESA values up to 20 m^2^/g were pointed out. Huang, J et al. [34] carried out a comparative study of the methods used to synthesize Pt_3_Co materials using sodium borohydride and hydrazine as the reducing agents, with the average particle size being equal to 15.9 and more than 50 nm, respectively, and ESA values of 16 and 14 m^2^/g, which were substantially lower than the ESA values for Pt/C (79 m^2^/g(Pt)), although the ORR activity and the stability of the Pt_3_Co material turned out to be higher than those of Pt/C. In their review article, Wenjuan Yan et al. [32] considered many other synthesis methods. It is worth noting that according to the review [32], the most commonly used precursors are platinum and cobalt acetylacetonates, H_2_PtCl_6_ and CoCl_2_, with sodium borohydride, ethylene glycol and 2-methylimidazole being used as the reducing agents. In addition to the synthesis methods, the authors paid particular attention to the shape and size of the NPs described in the literature. The analysis of the above data allowed them to draw the conclusion that the borohydride and polyol synthesis methods make it possible to obtain crystallites with the smallest particle size (≈2.5 nm) compared with the other ones considered in the review. Previously, we have proposed an alternative method to synthesize PtCo/C catalysts that differs from most of the one-step liquid-phase synthesis methods described in the literature due to the presence of a preliminary stage for the electrochemical preparation of a composite Co_x_O_y_/C support [40,41]. In this regard, the PtCo/C materials synthesized with the two-step method based on an electrochemically prepared composite Co_x_O_y_/C support with subsequent platinum deposition demonstrated up to two times greater specific catalytic ORR activity compared with commercial Pt/C analogs [41].

An important stage for the preparation of any bimetallic catalyst prior to its use in fuel cells is acid pretreatment, which allows for the synthesis of de-alloyed catalysts and prevents the poisoning of fuel cells with alloying metal ions [42,43,44].

We believe that the methods used to synthesize PtCo/C catalysts may be improved by using several synthesis stages, the combination of which would allow for the achievement of a synergistic effect and the development of an effective method for the synthesis of bimetallic catalysts with enhanced microstructure and, thus, higher catalytic activity and stability. We have proposed a new multi-step method for the synthesis of PtCo/C materials based on a Co_x_O_y_/C composite that combines the advantages of various wet-synthesis methods and allows for the preparation of materials with a high surface area and outstanding ORR activity.

## 2. Materials and Methods

### 2.1. Chemicals and Materials

Ketjenblack EC-300J as the carbon support, ethylene glycol (top grade, not less than 99.8%, Rehacor LLC, Moscow, Russia), H_2_PtCl_6_∙6H_2_O (TU 2612-034-00205067-2003, Pt mass fraction of 37.6%, Aurat, Moscow, Russia), CoSO_4_∙7H_2_O, sodium hydroxide (Rehacor LLC), argon (Ar, 99.9%, Globus, Moscow, Russia) and sulfuric acid (JSC Vekton, Saint Petersburg, Russia) were used in the experiment.

### 2.2. The Preparation of PtCo/C Catalysis by Multi-Step Synthesis

**Figure 1:**
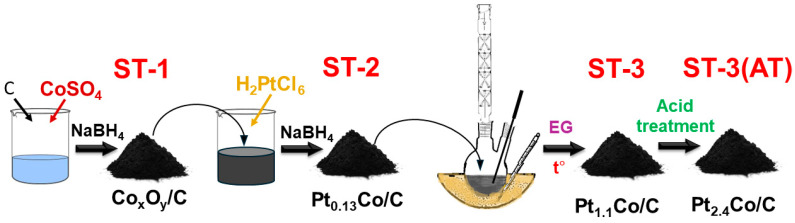
Scheme of multi-step synthesis for PtCo/C catalysts.

In this work, we propose a three-step synthesis method, which includes the stages of the borohydride and polyol synthesis methods (Figure 1).

The first stage is the preparation of a composite Co_x_O_y_/C support via the reduction of the cobalt precursor in an aqueous ethylene glycol suspension (the water–ethylene glycol ratio is 1:3) of the Ketjenblack EC-300J carbon support at a pH of 14 with a NaBH_4_ aqueous solution, which was added dropwise in a 10-fold excess with the constant stirring of the suspension. After adding the NaBH_4_ solution, the suspension was stirred on a magnetic stirrer for another 30 min, after which the resulting material was filtered using a Büchner funnel, rinsed repeatedly with bidistilled water and ethanol, and dried at a temperature of 70 °C under vacuum for 24 h.

The second stage is the deposition of platinum onto the composite support. The composite support prepared at the first stage was dispersed by ultrasound 3 times for 2 min (Soniprep ultrasonic homogenizer) in an aqueous ethylene glycol medium (the water–ethylene glycol ratio is 1:3), after which the calculated amount of the platinum precursor was added in order to have, with complete reduction, a 20% Pt mass fraction in the resulting material. Next, 10 mL of the aqueous solution containing a 10-fold excess of NaBH_4_ was added dropwise to the suspension with constant stirring, and the suspension was allowed to stand with constant stirring for another 30 min. The filtration and drying of the sample were carried out similarly to the first stage (as specified above).

The third stage is the deposition of platinum onto the PtCo/C catalyst with a 20% Pt mass fraction synthesized at the second synthesis stage. The PtCo/C catalyst synthesized at the second stage was dispersed by ultrasound 3 times for 2 min (Soniprep ultrasonic homogenizer) in ethylene glycol, after which the calculated amount of the platinum precursor in ethylene glycol (pH = 14) was added in order to have, with complete reduction, a 40% Pt mass fraction in the resulting material. Next, the suspension was transferred to a round-bottomed flask, which was placed in a sand furnace located on the magnetic stirrer with heating, and the reaction mixture was subjected to quick heating up to 160 °C with constant stirring at 500 rpm. After reaching the set temperature, the suspension was allowed to stand for 2 h at a constant temperature of 160 °C and with continuous stirring. After the synthesis was completed, the suspension was cooled to room temperature with stirring. The filtration and drying of the sample were carried out similarly to the first stage.

To conduct the acid treatment, the catalyst suspension was allowed to stand in an aqueous solution of 1 M nitric acid with constant stirring on the magnetic stirrer (500 rpm) for 3 h at room temperature. After the acid treatment, the catalyst suspension was filtered using the Büchner funnel, and the precipitate was subjected to threefold rinsing with bidistilled water and dried at a temperature of 70 °C under vacuum for 24 h.

### 2.3. Structural Study for Synthesized PtCo/C Catalysts

The mass fraction of metals in the samples synthesized at each stage was determined with thermogravimetry by the mass of the unburned residue. For this purpose, a weighed amount of the studied sample was placed into the calcined crucible with a constant mass and heat-treated at 800 °C for 40 min. After cooling, the crucible with the residue left after burning was weighed, with the mass fraction of the metals in the studied samples being calculated while taking into account the formation of Co_3_O_4_ oxide.

The Pt:Co ratio in the synthesized samples was determined with XRF analysis using an RFS-001 spectrometer (Research Institute of Physics, Southern Federal University, Rostov-on-Don, Russia). The analysis conditions were as follows: the voltage on the X-ray tube was 50 kV, the current was 150 μA, the anode’s material was molybdenum, and the spectrum acquisition time was 300 s. The X-ray fluorescence spectra were recorded and processed using the UniveRS 14_4 software (Southern Federal University, Rostov-on-Don, Russia).

The X-ray phase composition and the average crystallite size were determined with the XRD method using an ARL X’TRA powder diffractometer (Cu Kα). The analysis conditions were as follows: the experiment was conducted at room temperature, the angle range of 2θ was 15–55°. The average crystallite size was determined with the Scherrer equation [45]: D = Kλ/(FWHMcosθ), where K = 0.98 is the Scherrer’s constant, λ is the wavelength of the monochromatic radiation (in Å), FWHM is the reflex full width at half-maximum (in radians), D is the average crystallite size (in nm), and θ is the angle of incidence (in radians).

The size of the bimetallic NPs, as well as features of their size and spatial distributions, were studied with high-resolution transmission electron microscopy (HRTEM), HAADF-STEM and the Secondary Electron Imaging method (SEI). Micrographs were obtained using a JEOL JEM-F200 (JEOL, Tokyo, Japan) microscope (voltage 200 kV, current 12–15 μA, CFEG). The histograms of the NPs’ size distribution in the catalysts were plotted based on the results of the size determination for at least 300 particles randomly selected from the TEM micrographs at different sections of the sample. The error margin was ±0.2 nm.

### 2.4. Activity Study for Synthesized PtCo/C Catalysts

#### 2.4.1. Preparation of Catalytic Ink

The catalyst suspension (catalytic “ink”) was prepared by mixing 0.0040 g of the studied catalyst (for the catalyst with a Pt mass fraction ≈ 40%), 1700 µL of isopropyl alcohol, 1900 µL of deionized water and 400 µL of a Nafion^®^ D1021 1% aqueous emulsion. The suspension was then dispersed in an ultrasonic bath for 10 min and stirred using an INTLLAB laboratory vortex mixer (INTLLAB, Shanghai, China) for 1 min. After stirring, the suspension was dispersed in an ultrasonic bath for another 10 min and stirred again using the INTLLAB laboratory vortex mixer for 1 min. The water temperature in the ultrasonic bath was not to exceed 20 °C. The total preparation time for the homogeneous suspension was 30 min. Before applying the suspension, the RDE glass–carbon end face was polished using a polishing paste (allied aluminum oxide suspension). Next, in order to remove the paste residues from the electrode surface, the electrode was treated with ultrasound and rinsed with isopropyl alcohol. The prepared catalytic ink was used to apply a thin layer of the catalyst to the glass–carbon electrode.

#### 2.4.2. Formation of Catalytic Layer at RDE

A 5 µL aliquot of the catalytic ink was sampled with a pipette tip after stirring. A drop of the catalytic ink of the above volume was applied to the end face of the polished and degreased glass–carbon electrode with a diameter of 5 mm (area 0.196 cm^2^). After applying the ink drop, the electrode was dried at a rotation speed of 700 rpm to form a uniform catalytic layer. After drying the first drop of the catalytic ink, we applied the second one of the required volume calculated in order that the total volume of the applied ink was from 9 to 17 µL, depending on the Pt loading in the catalyst composition. The total Pt loading at the RDE end face was supposed to be 19.0–21.0 µg/cm^2^. Therefore, the studied electrode represented a homogeneous catalytic layer applied to the RDE.

#### 2.4.3. ESA Determination

The electrochemical measurements were performed in a three-electrode cell using a VersaSTAT potentiostat (AMETEK Scientific Instruments, Oak Ridge, TN, USA) and the rotating disk electrode (Pine Research Instruments, Durham, NC, USA). A saturated silver chloride electrode was used as the reference electrode. The potential values were given relative to the reversible hydrogen electrode (RHE) according to Formula (1):

E_RHE_ = E + E_SCE_ + E_pH_ − iR (1)

where

E_RHE_—potential value relative to the reversible hydrogen electrode (RHE), V;
E—is the set value of the potential, V;
E_SCE_—potential value of silver chloride electrode (Ag/AgCl), V;
E_pH_—correction for solution pH, for 0.1 M HClO4 equal to 0.059 V at 25 °C, V;
iR—is the ohmic potential drop equal to the product of the current strength and the resistance of the solution layer between the reference electrode and the studied one.

The surface activation was carried out in a three-electrode electrochemical cell. We used 0.1 M HClO_4_ as the electrolyte. Cyclic voltammograms (CVs) were recorded in the potential range of 0.04–1.00 V with a potential sweep rate of 100 mV/s during 100 cycles in an Ar atmosphere.

After the activation stage, the CVs were recorded in the potential range of 0.04–1.00 V with a potential sweep rate of 20 mV/s during 2 cycles. From the results obtained for the second CV, the ESA was calculated by the charge amount consumed for the desorption Q’ and adsorption Q” of hydrogen, as described in detail in [46].

#### 2.4.4. ORR Activity Determination

After measuring the ESA, the ORR activity of the catalyst at the RDE was estimated with linear sweep voltammetry. To assess the initial ORR activity of the catalysts, it was necessary to measure the background potentiodynamic polarization curve in an argon-saturated electrolyte at a rotation speed of 1600 rpm in the potential range of 0.05–1.1 V with a potential sweep rate of 20 mV/s. Next, the electrolyte in the cell was replaced with a freshly prepared one and saturated with oxygen for 1 h, after which the potentiodynamic curves were measured at a potential sweep rate of 20 mV/s in the potential range of 0.05–1.1 V at four RDE rotation speeds: 400, 900, 1600 and 2500 rpm. The potential was recalculated taking into account the resistance (iR compensation), the potential value of the reference electrode, and the pH value of the electrolyte solution. Using the Koutecký–Levich equation, the kinetic current values and the ORR parameters (mass activity and specific activity) were calculated, as described in [46].

#### 2.4.5. Assessment of Catalyst Stability

The stability of the catalysts was assessed with accelerated stress testing based on the repeated overlay of potential rectangular pulses of 0.4 and 1.0 V with an exposure time of 3 s at each potential value, as described in [47]. A total of 10,000 cycles were recorded. The measurements were carried out in the 0.1 M HClO_4_ solution saturated with oxygen at 25 °C. To study the degree of the degradation of the materials, CVs and linear sweep voltammograms (LSVs) were recorded before and after the stress testing, as described in Section 2.4.3 and Section 2.4.4. The degree of degradation (DD) was assessed by a change in the ESA and mass activity values upon the completion of the stress testing [46].

### 2.5. Activity Study for Catalysts in MEAs

#### 2.5.1. Preparation of Catalysts for MEAs

For the preparation of the catalytic ink, a suspension of the corresponding Pt/C or PtCo/C catalyst and a calculated volume of Nafion^®^ D1021 were dispersed in deionized water and isopropyl alcohol in a ratio of 2:1, respectively. The Nafion solution was added in a volume calculated in order to have a 7:10 ratio of Nafion:carbon. The catalytic ink was dispersed in an ultrasonic bath 3 times for 20 min with intermittent stirring using the INTLLAB laboratory vortex mixer for 1 min. The ink was then sprayed with a spraying gun directly onto the Nafion 212 membrane with an active area of 5 cm^2^, which was placed on a heating surface at a temperature of 90 °C. For the catalytic ink forming the cathode, the Pt loading was 0.3 mgPt cm^−2^; for the anode, it was 0.4 mgPt cm^−2^. A commercial JM40 sample was used as the anode catalyst for all MEAs.

#### 2.5.2. Preparation of MEAs

All MEAs were formed by hot pressing. For this purpose, a membrane with the sprayed catalytic layers (catalyst coated membrane) was placed between two sheets of gas diffusion layers (Toray TGP-H-060). The pressing was carried out at a temperature of 130 °C and a pressure of 80 kgf cm^−2^ for 3 min. After that, the resulting MEA was left to cool at room temperature for 15 min.

#### 2.5.3. Testing of Single Fuel Cells

The testing of single fuel cells was carried out using a BioLogic FCT-50S (Grenoble, France) test station in a single PaxiTech (Échirolles, France) cell with an active area of 5 cm^2^. Hydrogen (99.999% pure) was used as the gas supplied to the anode, and air with no mechanical impurities was used at the cathode. The temperature of a single fuel cell was 80 °C. The humidity of the gases was 100%, and the gas flow was constant and amounted to 220 mL min^−1^ for hydrogen and 680 mL min^−1^ for air. The back pressure of the gases was 1.5 bar. The activation of an MEA as part of a single fuel cell was carried out by holding at 0.6 A cm^−2^ until the voltage change was ±5 mV for 15 min. The discharge curves of the MEA were measured in the range of 0.1–2 A cm^−2^, with the automatic shutdown of the fuel cell at a voltage drop below 0.2 V.

## 3. Results

### 3.1. Structural Characteristics of PtCo/C Catalysts Synthesized with Three-Step Method

**Figure 2:**
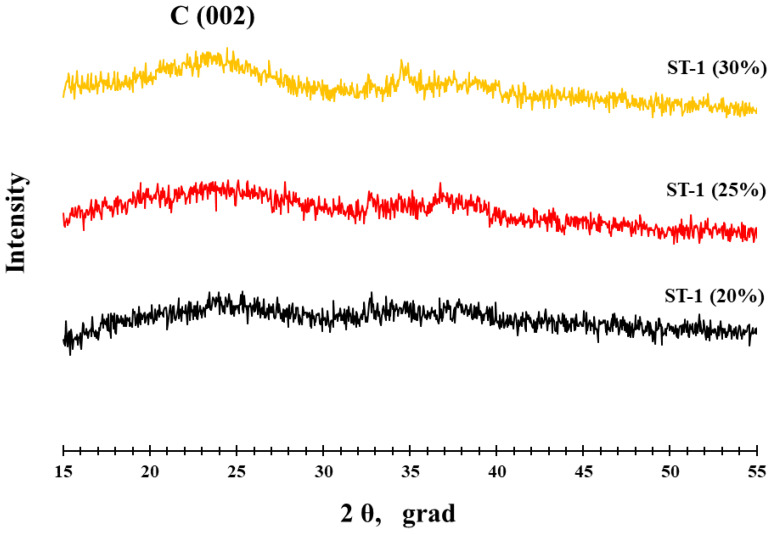
X-ray diffraction patterns of the materials: Co_x_O_y_/C—20%; Co_x_O_y_/C—25%; Co_x_O_y_/C—30%.

The study of the materials’ composition and structure at each stage of the synthesis (Figure 2) confirmed a gradual increase in the mass fraction of metals step-by-step up to 52.9% and an increase in the platinum content at each stage (Table 1) up to the Pt1.1Co composition. The X-ray powder diffraction (XRD) analysis of the ST-1 material synthesized at the first stage (Figure 2) showed the presence of a poorly pronounced carbon phase, with reflections corresponding to metallic cobalt or its oxides not being observed (Figure 2). Taking into account the results of the gravimetric and elemental analyses (Table 1) that demonstrated the presence of cobalt in the material, it can be assumed that in this case, cobalt was present in the material in the form of X-ray amorphous oxide. 

**Figure 3:**
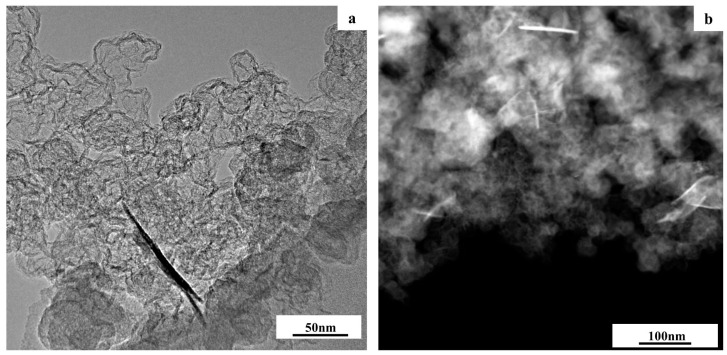
TEM (**a**) and HAADF STEM (**b**) micrographs of the Co_x_O_y_/C ST-1 material.

**Figure 4:**
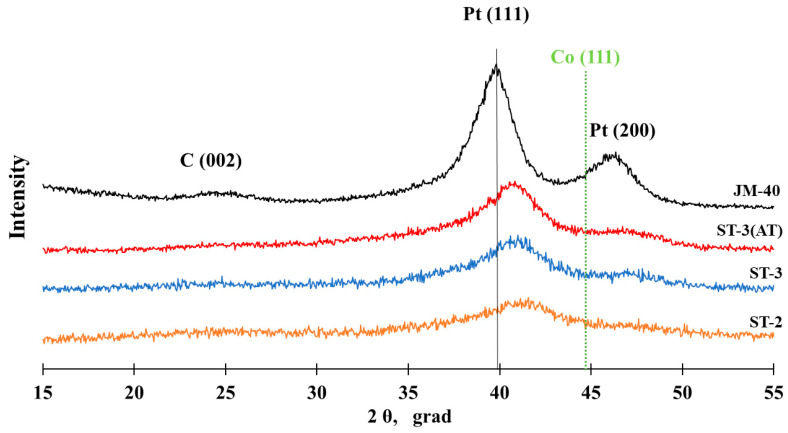
X-ray diffraction patterns of the PtCo/C and commercial Pt/C JM40 materials.

The study of the ST-1 material with the transmission electron microscopy (TEM) method (Figure 3) confirmed the formation of cobalt oxide in the form of elongated filamentous structures shaped like “leaf-shaped” with a thickness of about 5–10 nm and a length of about 100–200 nm on the carbon support surface. The results of the line scanning of the elemental composition (Figure S1) and the elemental mapping of a separate section of the material surface (Figure S2) confirmed that the individual structures contained a large amount of cobalt. It is also noteworthy that varying the mass fraction of cobalt in the materials from 20 to 30% at the first synthesis stage failed to lead to any additional reflections in the X-ray patterns, as all the XRD spectra were identical (Figure 2). After the deposition of platinum at the second synthesis stage, the reflections corresponding to a face-centered cubic (FCC) structure with a crystal lattice parameter of 3.748 Å could be observed in the X-ray pattern of the ST-2 material (Figure 4) in addition to the reflections of the carbon support C (002) at about 25° 2θ. It should be pointed out that the lattice parameter for the ST-2 material was significantly inferior to the platinum lattice parameter (3.850 Å), which indicates the formation of a PtCo solid solution. Indeed, a comparison of the X-ray patterns for the synthesized ST-2 material and the commercial Pt/C catalyst demonstrates a substantial shift in the reflections to the high-angle region of 2θ for the ST-2 material compared with Pt/C. The calculation of the PtCo solid solution composition according to Vegard’s law [49] for the ST-2 material exhibited the composition of Pt1.3Co (Table 1), which significantly differed from the total composition of the ST-2 material based on the X-ray fluorescence (XRF) analysis. This fact may be related to the incomplete entry of cobalt into the composition of the Pt–Co bimetallic solid solution, with the rest of the cobalt being present in the form of X-ray amorphous oxide. Similar to the X-ray pattern of the commercial Pt/C catalyst, for the reflections of the ST-2 material metal phase, we observed a pronounced broadening of reflections in the X-ray pattern, which is associated with the synthesis of metal nanodispersed particles, the average crystallite size of which can be estimated using the Scherrer equation [45]. The estimation of the average crystallite size for the ST-2 material confirmed the formation of bimetallic NPs with an average size of about 1.4 nm on the surface of a highly dispersed carbon support, the size of the metal NPs being significantly smaller compared with the size of the platinum NPs for the commercial Pt/C material (Table 1). It should be noted that the decrease in crystallite size compared with the Pt/C materials is typical for bimetallic NPs and may be due to the alloying of platinum with various d-metals [50].

The further deposition of platinum at the third synthesis stage resulted in a shift in the reflection maxima of the metal phase for the ST-3 sample to the low-angle region of 2θ compared with the ST-2 material, which correlated well with an increase in the platinum content in the material. Similarly to the ST-2 material, the inconsistency between the total composition of the ST-3 material according to total reflection X-ray fluorescence (TXRF) (Pt_0.9_Co) and the solid solution composition for the bimetallic NPs determined with Vegard’s law (Pt_2.4_Co) may have been due to the presence of X-ray amorphous cobalt oxide in the material, which was not included in the solid solution with platinum. It is noteworthy that the average crystallite size for the ST-3 material increased by 0.4 nm compared with the ST-2 material, which may have been due to the platinum deposition and an increase in the mass fraction of metals in the material. To prevent the dissolution of the alloying component during the operation of the catalyst in fuel cells, the resulting ST-3 material was treated with acid, as a result of which the ST-3(AT) material with an atomic metal ratio of Pt_2.4_Co was synthesized. The change in the composition of the ST-3(AT) material appears to have been due to the dissolution of cobalt, which was not included in the solid solution with platinum. It should be pointed out that the composition of the metal phase calculated according to Vegard’s law for the ST-3(AT) material also differed from the composition determined from the TXRF data, which may indicate the dissolution of cobalt from the surface of the bimetallic NPs. The average crystallite size for the material after the acid treatment increased by 0.8 nm, which may have been due to the dissolution of X-ray amorphous cobalt oxide, the dissolution of cobalt, and the detachment of the smallest NPs from the surface of the carbon support.

**Figure 5:**
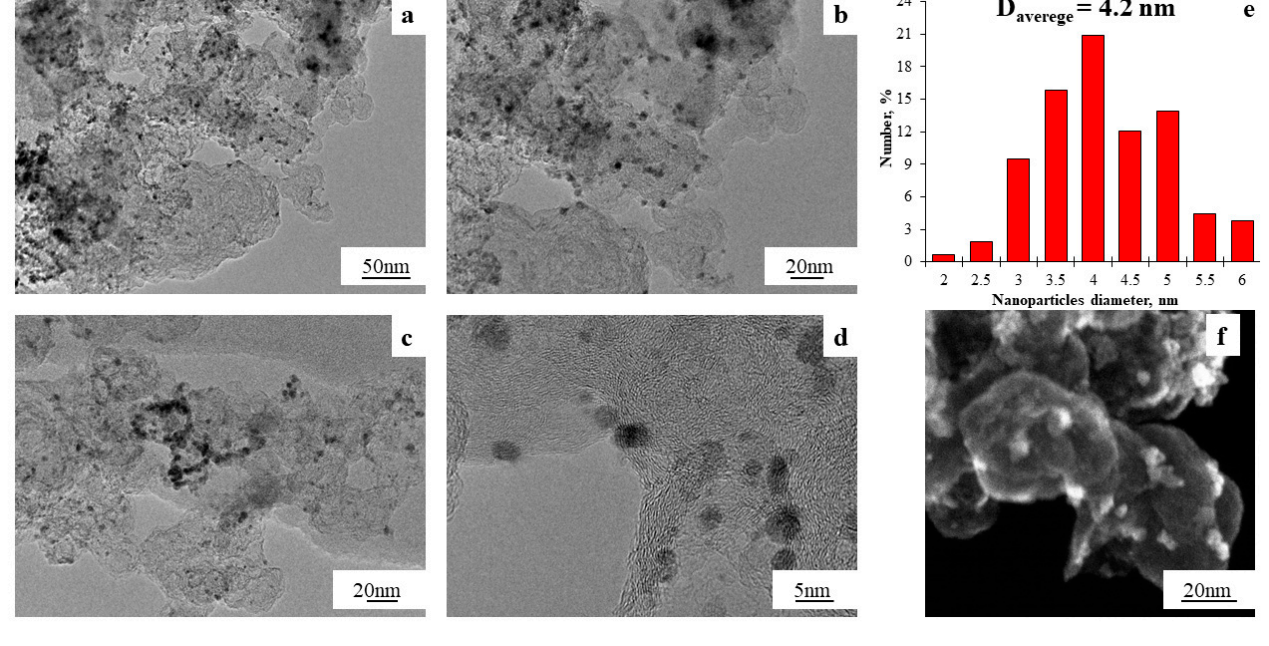
TEM micrographs (**a**–**d**) and histograms of the PtCo NP size distribution (**e**) and Secondary Electron Imaging (SEI) (**f**) for the PtCo/C ST-2 material.

The microstructure of the PtCo/C materials synthesized at various stages was additionally studied with TEM. The ST-2 material was characterized by the formation of metal NPs on the surface of the carbon support (Figure 5a–d) with a wide NP size dispersion from 2 to 6 nm (Figure 5e) and an average NP size of 4.2 nm, which significantly exceeded the average crystallite size for this material. The larger average size of NPs determined with the TEM method, compared with the average crystallite size calculated from the XRD data, is typical for platinum-based catalysts [51] and may be associated both with the presence of a disordered surface layer of metal NPs and with the entry of several crystallites into the composition of an individual NP. The line scanning of the elemental composition for a separate surface section of the ST-2 material (Figure S3) confirmed the formation of bimetallic NPs.

**Figure 6:**
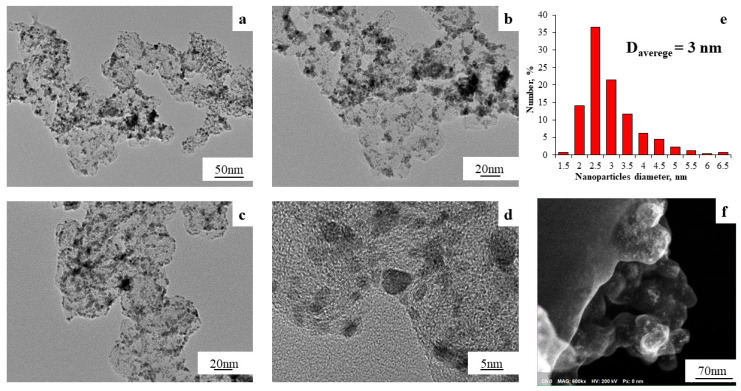
TEM micrographs (**a**–**d**), histograms of the PtCo NP size distribution (**e**) and Secondary Electron Imaging (SEI) (**f**) for the PtCo/C ST-3 material.

For the PtCo/C ST-3 material, using the TEM method, we observed the formation of bimetallic NPs on the surface of the carbon support (Figure 6a–d) with a high proportion of particles of about 2.5 nm in size (Figure 6e) and an average NP size of 3.0 nm, which was significantly lower than the average NP size for the ST-2 material. This fact may be related to differences in the synthesis methods at each stage, as the polyol method was used at the third stage, which allowed for the synthesis of smaller particles and due to which the average size of NPs in the ST-3 material decreased compared with the ST-2 material synthesized with the borohydride method. For the ST-3 material, the line scanning of the elemental composition also confirmed the formation of bimetallic particles (Figure S4), as in the case of the ST-2 material.

The TEM study of the ST-3(AT) material after the acid treatment demonstrated insignificant differences in the microstructure of this catalyst (Figure S6) compared to the ST-3 material before the treatment, which confirmed a slight change in the average size of the ST-3(AT) catalyst NPs up to 2.7 nm (Table 1) and which may have been due to the dissolution of cobalt, including that from the surface of the bimetallic NPs.

### 3.2. Catalytic Activity of PtCo/C Catalysts Synthesized with Three-Step Method

The cyclic voltammograms recorded during the electrochemical surface activation for the PtCo/C materials synthesized at various stages and the commercial Pt/C catalyst (Figure S6) showed no maxima corresponding to the electrochemical dissolution of metals. At the same time, due to the development and cleaning of the current surface, the appearance of the CVs changed slightly until the tenth–twentieth cycle, after which there were no changes observed in curves from cycle to cycle, the latter indicating the successful standardization of the materials.

**Figure 7:**
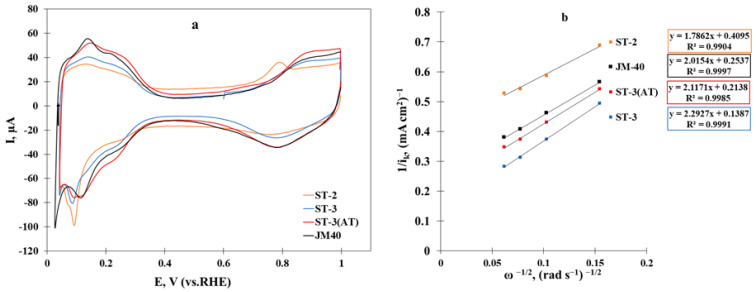
(**a**) Cyclic voltammograms (2nd cycle). Electrolyte 1 M HClO_4_, Ar atmosphere. The potential scanning rate was 20 mV/s. (**b**) K–L plots. The rotation speeds were 400, 900, 1600, and 2500 rpm, and the potential sweep rate was 20 mV/s. Electrolyte 0.1 M HClO_4_, O_2_ atmosphere.

The CVs for the samples after activation have a characteristic appearance for platinum-based electrocatalysts [9,21,24]. In all the CVs, we could observe characteristic regions, i.e., the hydrogen one, in which the processes of hydrogen adsorption and desorption proceed (0.04–0.30 V); the region corresponding to the non-faradaic processes of the electrical double layer charge and discharge (0.30–0.60 V); and the oxygen region, in which the processes of oxygen adsorption and desorption proceed (0.60–1.00 V). The CV appearance for the PtCo/C ST-2 catalyst (Figure 7a) differed significantly compared with the other studied materials. The voltammogram of the ST-2 sample was characterized by higher currents in the double-layer region, which may have been due to the high porosity of the KetjenBlack EC-300J carbon support used. It should be noted that the CV of the ST-1 material (Figure S7) exhibited no maxima of hydrogen adsorption and desorption and platinum oxidation/reduction, since the material only contained X-ray amorphous cobalt oxide on the carbon support. At the same time, similar to the ST-2 material, the CV for ST-1 was also characterized by higher currents in the double-layer region, which may have been due to the high proportion of a highly dispersed carbon support. When further synthesizing the ST-3 and ST-3(AT) materials, as a result of doubling the amount of the metal component, the contribution of the support to the electric double layer capacitance appeared to decrease, which was reflected in a decrease in the double-layer region currents. An additional peak in the potential range of 0.7–0.8 V can be observed in the CV of the ST-2 material, which may be associated with the presence of cobalt oxide [41]. The ESA value varied in the following order: ST-2 < ST-3 ≤ JM40 < ST-3(AT) (Table 2). The higher ESA values of the ST-3 catalyst (55 m^2^/g(Pt)) compared with ST-2 (37 m^2^/g(Pt)), despite the increase in the platinum loading, can be explained by the dimensional effect, which is associated with synthesis specifics. The ST-2 material was synthesized with the borohydride method, which resulted in the appearance of larger NPs of more than 3 nm in size (Figure 5) [34]. The polyol synthesis method, on the contrary, allowed for the synthesis of NPs of a smaller size (less than 3 nm), as can be seen in Figure 6 [36]. Due to the predominant proportion of these particles in the ST-3 sample, the average NP size in the ST-3 material was smaller than that in the ST-2 material (Table 1). The ESA value of the ST-3(AT) material increased compared with ST-3 due to the selective dissolution of cobalt from the surface of the bimetallic NPs as a result of the acid treatment. It is worth noting that the ESA values of the ST-3(AT) and ST-3 catalysts are comparable to the commercial Pt/C analog (Table 2). Therefore, the multi-step synthesis method, which combines the advantages of various liquid-phase synthesis methods, makes it possible to synthesize PtCo/C materials with higher ESA values, which is quite difficult for bimetallic catalysts [34].

The estimation of the catalytic ORR activity for the synthesized materials with linear sweep voltammetry at the rotating disk electrode (RDE) with different rotation speeds was carried out by determining the kinetic current at a potential of 0.9 V according to the Koutecký–Levich equation (Figure 7b). It should be pointed out that the activity values for ST2 and ST3 changed similarly to the change in the ESA values (Table 2). As a result, the PtCo/C material synthesized with the two-step method demonstrated a 1.75 times greater mass and specific ORR activity compared with its commercial analog. Despite the increase in the ESA values as a result of the acid treatment, the mass activity of the ST-3(AT) material decreased by 1.75 times compared with ST-3. This result is in good agreement with the literature data [47,52]. Nevertheless, the material after the acid treatment exhibited activity values that were comparable to the commercial Pt/C material.

**Figure 8:**
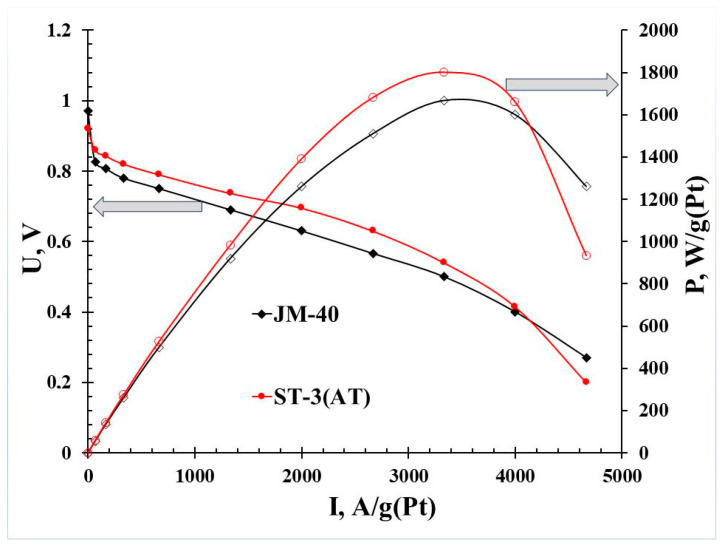
Polarization plots of MEAs with an ultralow platinum group metals (PGMs) loading (total loading (anode + cathode) of 0.070 mg_PGM_ cm^−2^ including both the cathode and anode) tested in H_2_/air. Comparison of Pt/C and PtCo/C.

For the synthesized PtCo/C catalyst after the acid treatment and its commercial analog JM40, a comparative study of the activity at the MEA cathode was carried out. According to the results of the analysis of the obtained current–voltage characteristics (Figure 8), the activity of the PtCo/C catalyst synthesized with the multi-step method exceeded that of the commercial Pt/C analog, e.g., at low current densities (0.4 A/cm^2^), the voltage in the MEA at the cathode of which the ST-3(AT) material was used was 50 mV higher than that in the MEA where the Pt/C catalyst JM40 was used. The maximum voltage differences for both studied materials were observed at a current density of 0.6 A/cm^2^, i.e., for the ST-3(AT) material, the MEA voltage at the above current density was 70 mV higher. At high current densities (1.2 A/cm^2^), the voltage in the MEA at the cathode of which the ST-3(AT) material is used was 20 mV higher compared with the Pt/C catalyst JM40. Therefore, the PtCo/C catalyst demonstrated the best characteristics in the entire studied current range. The maximum specific power of the sample with the ST-3(AT) material in the composition was 540 mW/cm^2^ (1800 W/g(Pt)), while the sample with JM40 at the cathode exhibited a maximum specific power of 500 mW/cm^2^ (1667 W/g(Pt)).

**Figure 9:**
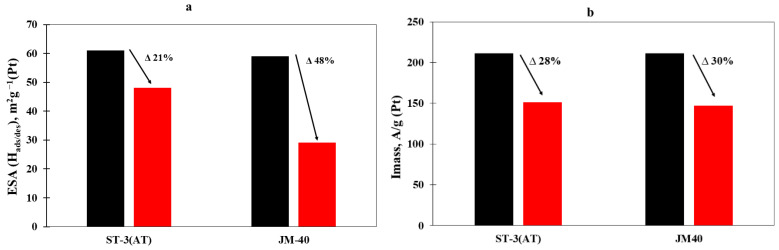
Histograms of the (**a**) ESA and (**b**) mass activity changes before (black column) and after (red column) stress testing.

The stability study of catalysts is an important stage in estimating the characteristics of their electrochemical behavior, since catalyst stability largely determines a fuel cell’s service life [53]. The stability of the ST-3(AT) and JM40 materials was estimated with the method of the repeated sequential overlay of potentials of 0.4 and 1.0 V during 10,000 cycles (see measurement procedure). The material degradation was calculated via changes in the ESA and ORR activity values. Though it was established that the studied catalysts had close initial ESA values (Figure 9a), after the stress testing, decreases in ESA were observed to varying degrees. For example, for the PtCo/C ST-3(AT) catalyst after the stress testing, the change in the ESA was observed to a lesser extent of up to 48 m^2^/g(Pt) and only comprised 21% of the initial value, while for the commercial Pt/C material, the degree of ESA degradation was twice as high, with the ESA value after the stress testing amounting to 29 m^2^/g(Pt) (Figure 9a).

Additionally, the change in the materials mass activity determined at a potential of 0.9 V after the stress testing was studied. The mass activity values of the ST-3(AT) and JM40 catalysts after the stress testing were close enough, amounting to 151 and 147 A/g(Pt), respectively. The degradation of the PtCo/C material synthesized with the multi-step method was comparable in activity to the commercial catalyst, amounting to about 30% (Figure 9b).

## 4. Conclusions

The results of this study show that a multi-step synthesis method based on a composite carbon oxide support allows for the synthesis of PtCo/C catalysts with bimetallic NPs of an average 2.7 nm in size and which are uniformly distributed over the surface of the carbon support due to a combination of the advantages of different liquid-phase synthesis methods. The synthesized PtCo/C catalysts were characterized by a high active surface area and a specific ORR activity 1.75 times higher than that of a commercial Pt/C material. The acid treatment of the synthesized PtCo/C material was conducive to the dissolution of cobalt in a composition of Pt_2.4_Co and a decrease in catalytic ORR activity. However, this notwithstanding, the material after acid treatment was not inferior to the commercial Pt/C catalyst in its activity and stability. It is obvious that the de-alloying methods for PtCo/C catalysts require further elaboration, which will be the subject of further research. At the same time, according to the results of a comparative study for the activity of an ST-3(AT) material at an MEA cathode, it was shown to demonstrate better current–voltage characteristics at 40 mW/cm^2^ and a higher maximum specific power compared with the MEA at the cathode of which the commercial Pt/C catalyst was used, which confirms the prospects for the proposed method to synthesize bimetallic PtCo/C catalysts. The developed novel approach to synthesizing bimetallic catalysts may enhance the activity of PtM/C catalysts and reduce the specific platinum loading in their catalytic layer, which would contribute to improving the consumer properties of PEMFCs.

## Figures and Tables

**Table 1:** Some parameters of the composition and structure of the PtCo/C and commercial Pt/C materials.

Designation	Chemical Composition (Theoretical)	Chemical Composition (XRF)	Chemical Composition (XRD)	Pt Mass Fraction (Theoretical), %	Mass Fraction of Metals (Gravimetry), %	Average Crystallite Size (XRD), nm	Average Size of Bimetallic NPs (TEM), nm	Lattice Parameter, Å
ST-1	Co	Co	-	-	24.9	-	-	-
ST-2	PtCo_4.1_	PtCo_7.4_	Pt_1.3_Co	20	45.6	1.4	4.2	3.748
ST-3	Pt_1_Co	Pt_0.9_Co	Pt_2.4_Co	40	52.9	1.8	3.0	3.815
ST-3(AT)	Pt_1_Co	Pt_2.4_Co	Pt_3.1_Co	40	-	2.6	2.7	3.836
JM40	Pt	Pt	Pt	40	40	3.2	3.3 [48]	3.923

**Table 2:** Some parameters of the catalytic activity for the PtCo/C and commercial Pt/C materials.

Material	ESA, m^2^/g(Pt)	I_k_, mA	I_mass_, A/g(Pt)	I_spec_, A/m^2^(Pt)
ST-2	37	0.48	130	3.5
ST-3	55	1.42	369	6.7
ST-3(AT)	61	0.92	211	3.5
JM40	56	0.82	211	3.8

## Data Availability

Data are contained within the article.

## Supplementary Materials

The following supporting information can be downloaded at: https://www.mdpi.com/article/10.3390/nano14100856/s1, Figure S1: Line scanning for the ST-1 material; Figure S2: Element mapping of a separate section of the ST-1 material surface; Figure S3: Line scanning for the ST-2 material; Figure S4: Line scanning for the ST-3 material; Figure S5: TEM micrographs and histograms of the PtCo NP size distribution for the PtCo/C ST-3(AT) material; Figure S6: Cyclic voltammograms (100 cycles) for the PtCo/C catalysts ST-2, ST-3, and ST-3(AT) and the Pt/C catalyst JM40. Electrolyte 1 M HClO_4_, Ar atmosphere. The potential scanning rate was 20 mV/s; Figure S7: (a) Cyclic voltammograms (2nd cycle) for the Co_x_O_y_/C ST-1 material. Electrolyte 1 M HClO_4_, Ar atmosphere. The potential scanning rate was 20 mV/s.
