# Supplementary material for: New Approach to Synthesizing Cathode PtCo/C Catalysts for Low-Temperature Fuel Cells
Source: Nanomaterials (Basel). 2024 May 14;14(10):856. doi: 10.3390/nano14100856 (PMC11124439; doi:10.3390/nano14100856)
Supplement: Supplementary file 1 [file nanomaterials-14-00856-s001.zip › nanomaterials-3014680-supplementary.pdf]

Supplementary Materials

New Approach to Synthesizing Cathode PtCo/C Catalysts for Low-Temperature Fuel Cells

Sergey Belenov [1,2,*], Dmitriy Mauer [1,2], Elizabeth Moguchikh [1,2], Anna Gavrilova [1], Alina Nevelskaya [1,3], Egor Beskopylny [1,2], Ilya Pankov [4], Aleksey Nikulin [3], Anastasia Alekseenko [1,2]

1 Faculty of Chemistry, Southern Federal University, 7 Zorge St., Rostov-on-Don 344090, Russia; dima333000@yandex.ru (D.M.); liza.moguchix@mail.ru (E.M.); agavrilo@sfedu.ru (A.G.); alya.nevelskaya@mail.ru (AN); gosha200225@yandex.ru (E.B.); an-an-alekseenko@yandex.ru (A.A.)
2 Prometheus R&D LLC, 4G/36 Zhmaylova St., Rostov-on-Don 344091, Russia
3 Federal Research Center "The Southern Scientific Center of the Russian Academy of Sciences" (SSC RAS), Federal State Budgetary Institution of Science, 41 Chekhova St., Rostov-on-Don 344006, Russiaa; ynikulin@sfedu.ru
4 Research Institute of Physical Organic Chemistry, Southern Federal University, 194/2 Stachki St., Rostov-on-Don 344090, Russia; ipankov@sfedu.ru
* Correspondence: sbelenov@sfedu.ru or serg1986chem@mail.ru

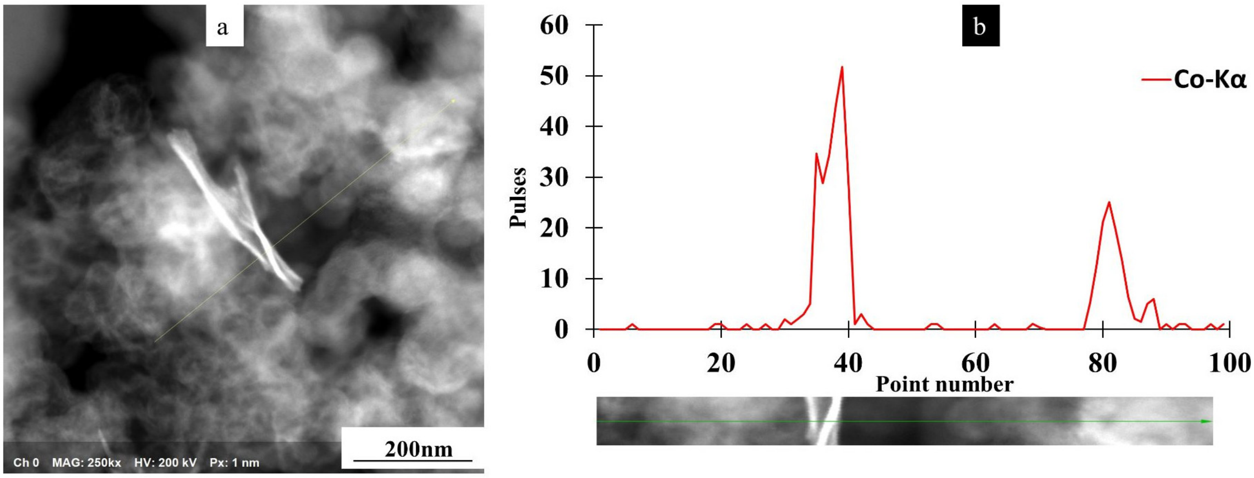

Figure S1. Line scanning for the ST-1 material.

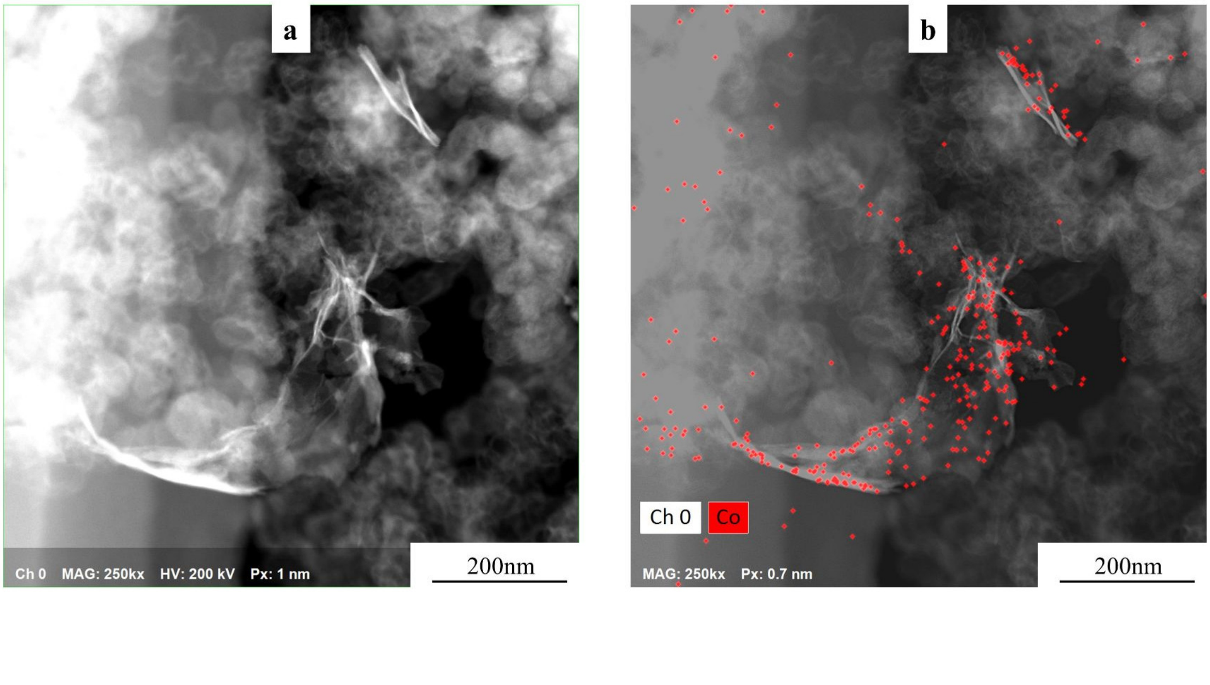

Figure S2. Element mapping of a separate section of the ST-1 material surface.

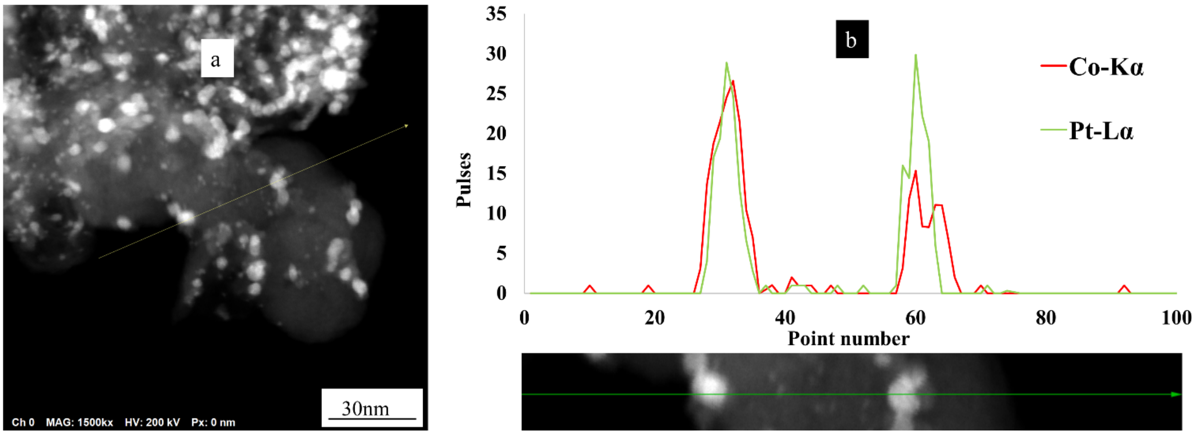

Figure S3. Line scanning for the ST-2 material.

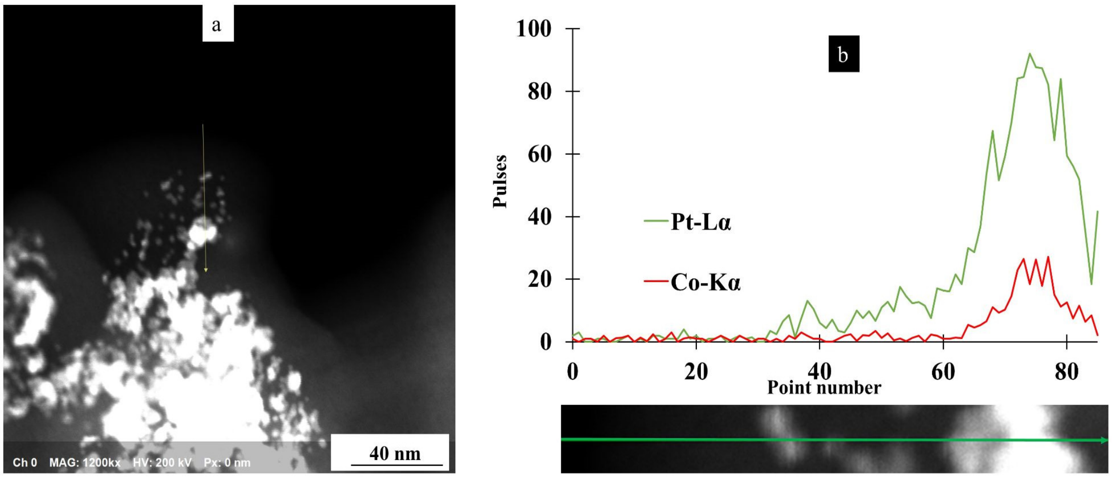

Figure S4. Line scanning for the ST-3 material.

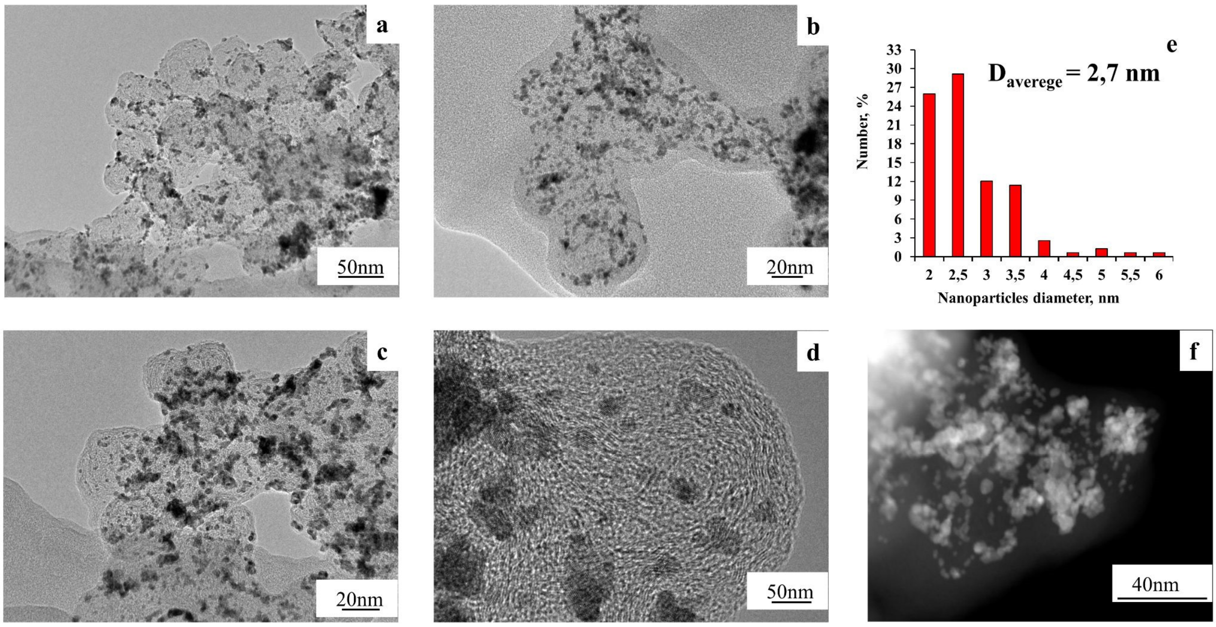

Figure S5. TEM micrographs and histograms of the PtCo NPs size distribution for the PtCo/C ST-3(AT) material.

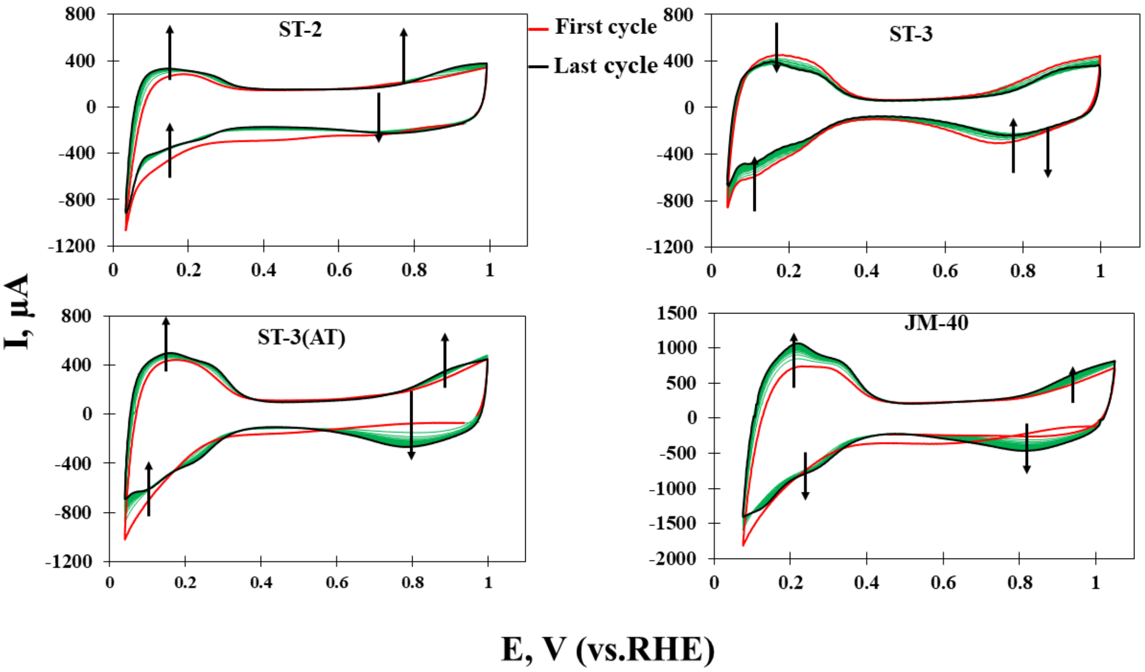

Figure S6. Cyclic voltammograms (100 cycles) for the PtCo/C catalysts ST-2, ST-3, ST-3(AT) and the Pt/C catalyst JM40. Electrolyte 1 M $HClO_4$, Ar atmosphere. The potential scanning rate is 20 mV/s.

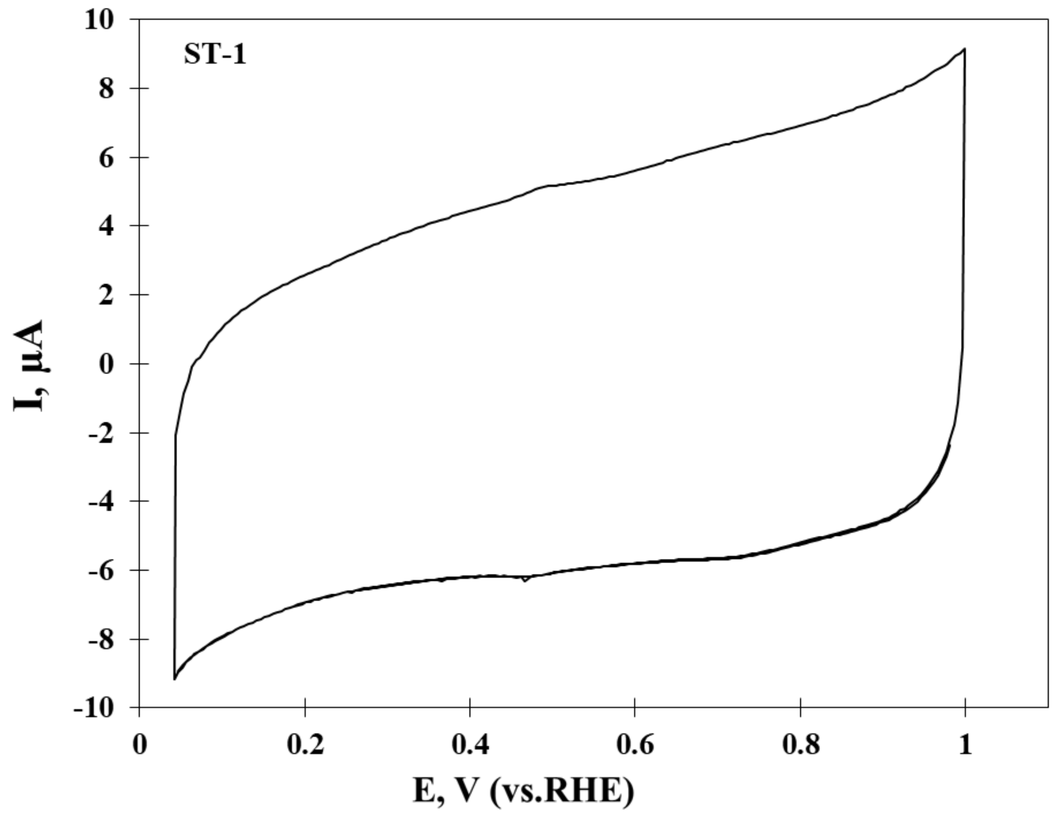

Figure S7. (a) Cyclic voltammograms (2nd cycle) for the Co_xO_y/C ST-1 material. Electrolyte 1 M $HClO_4$, Ar atmosphere. The potential scanning rate is 20 mV/s.
